# Long non-coding RNA UBE2CP3 enhances HCC cell secretion of VEGFA and promotes angiogenesis by activating ERK1/2/HIF-1α/VEGFA signalling in hepatocellular carcinoma

**DOI:** 10.1186/s13046-018-0727-1

**Published:** 2018-06-04

**Authors:** Jinduan Lin, Shunwang Cao, Yu Wang, Yanwei Hu, Hongwei Liu, Jiehua Li, Jing Chen, Pan Li, Jumei Liu, Qian Wang, Lei Zheng

**Affiliations:** 1 Department of Laboratory Medicine Center, Nanfang Hospital, Southern Medical University/The First School of Clinical Medicine, Southern Medical University, Guangzhou, Guangdong 510515 China; 2 Department of Clinical Laboratory, The Sixth Affiliated Hospital of Guangzhou Medical University, Qingyuan People’s Hospital, Qingyuan, Guangdong China; 3 Department of Hepatobiliary Surgery, Nanfang Hospital, Southern Medical University/The First School of Clinical Medicine, Southern Medical University, Guangzhou, Guangdong China

**Keywords:** Long non-coding RNA, UBE2CP3, HCC, Angiogenesis, ERK, VEGFA, Co-culture

## Abstract

**Background:**

Angiogenesis is considered as an important process in the development of malignancies and is associated with cancer progression and metastasis. Hepatocellular carcinoma (HCC) is the most common primary tumor of the liver and is recognized as a typical angiogenic tumor. Thus, it is of great importance to study the underlying mechanism of angiogenesis in HCC. The long non-coding RNA (lncRNA) ubiquitin conjugating enzyme E2C pseudogene 3 (UBE2CP3) has been reported as an oncogene that promotes tumor metastasis in HCC. However, the role and underlying mechanisms of UBE2CP3 in HCC angiogenesis are still unclear.

**Methods:**

We measured the expression levels of UBE2CP3 by in situ hybridization (ISH) and quantitative real-time polymerase chain reaction (qRT-PCR) in HCC patient samples. We also concomitantly used CD31/PAS double-staining to measure endothelial vessel (EV) density and used qRT-PCR to measure the CD31 mRNA level. HepG2 and SMMC-7721 cells were transfected with Lv-UBE2CP3 or Sh-UBE2CP3 virus to obtain stably over-expressing or knocking-down UBE2CP3 cell lines. The indirect effects of UBE2CP3 on ECs were studied by establishing a co-culture system using Transwell chambers with a 0.4-μm pore size. HCC cells and ECs in the co-culture system were separated, but the cytokines and growth factors were able to communicate with each other. Following exposed to HCC cells, ECs were collected for functional studies. Finally, we studied the function of UBE2CP3 in vivo by chick embryo chorioallantoic membrane (CAM) angiogenesis assays and nude mouse tumorigenicity assays.

**Results:**

In this study, we found that UBE2CP3 expression was higher in HCC tissues than in para-tumor tissues and was up-regulated in tissues with high EV density. Functionally, we found that in the co-culture systems, HCC cells overexpressing UBE2CP3 promoted HUVEC proliferation, migration and tube formation via the activation of ERK/HIF-1α/p70S6K/VEGFA signalling, increasing the level of VEGFA in HCC cell supernatant. In addition, the opposite results appeared when the expression of UBE2CP3 in HCC cells was knocked down. Consistent with these results, CAM angiogenesis assays and nude mouse tumorigenicity assays showed that UBE2CP3 expression up-regulated EV density in vivo.

**Conclusion:**

Our study suggests that UBE2CP3 can enhance the interaction between HCC tumor cells and HUVECs and promote HCC tumorigenicity by facilitating angiogenesis.

**Electronic supplementary material:**

The online version of this article (10.1186/s13046-018-0727-1) contains supplementary material, which is available to authorized users.

## Background

Hepatocellular carcinoma (HCC) is one of the most common malignancies and is known for its poor prognosis [1]; worldwide, it is the second and sixth leading cause of cancer-related death in men and women, respectively [2]. Therefore, it is important to explore the underlying mechanisms involved in the pathogenesis of HCC. It is well-established that HCC, especially moderately to poorly differentiated HCC, is a typical angiogenic tumor [3]. An increasing number of studies have verified that the dysregulation of angiogenesis is associated with cancer progression and metastasis [4]. As such, studies on the molecular mechanism of angiogenesis in HCC are greatly needed.

Angiogenesis is the formation of new vessels from pre-existing vasculature [5]. More and more studies suggest that angiogenesis plays a key role in cancer development. To date, anti-angiogenic therapy has become to be one of the anti-cancer strategies. A considerable number of anti-angiogenic drugs have been approved by the FDA and have been used in cancer treatment. Angiogenesis has been confirmed as a complex process that involves multiple steps such as the degradation of the basement membrane near the original vessels and endothelial cell (EC) proliferation, migration, aggregation and new tube formation, which eventually forms a new blood vessel system. Tumor angiogenesis involves interactions among tumor cells, ECs and mesenchymal cells through growth factors or cytokines and their corresponding receptors. Among these growth factors, vascular endothelial growth factor A (VEGFA) plays a key role in the pathophysiological process of angiogenesis [6]. Normally, the secretion levels of VEGFA are strictly controlled; when the levels of VEGFA are dysregulated, abnormal vessel formation occurs. Recently, studies have shown that many cancer cells can upregulate the expression of VEGFA to promote tumor angiogenesis [7]. However, the upstream mechanisms in VEGFA expression regulations are still incompletely understood.

Long non-coding RNAs (lncRNAs) are defined as a kind of RNA that is more than 200 nucleotides long and that lacks an open reading frame (ORF) with little or no protein-coding capacity. Multiple studies have shown that lncRNA participates in essential physiological and pathological processes including tumorigenesis and tumor progression [8–12]. Furthermore, lncRNAs have been reported to play a regulatory role in tumor angiogenesis [13, 14]. The lncRNA ubiquitin conjugating enzyme E2C pseudogene 3 (UBE2CP3) is recognized as an oncogene and has been reported to promote tumor metastasis by inducing epithelial–mesenchymal transition (EMT) in HCC [15]. It was recently reported that EMT can promote tumorigenicity in breast cancer cells by increasing tumor angiogenesis [16]. Therefore, we hypothesized that UBE2CP3 may play a major role in angiogenesis.

In this study, we measured endothelial vessel (EV) density by anti-CD31 immunohistochemistry (IHC) and quantitative real-time polymerase chain reaction (qRT-PCR). We analysed the expression of UBE2CP3 by qRT-PCR and in situ hybridization (ISH). Our results showed that UBE2CP3 was frequently highly expressed in HCC tissues, especially in high EV density HCC tissues. To study the indirect effects of UBE2CP3 in ECs, we constructed a co-culture system to simulate the internal interaction by using Transwell chambers with a 0.4-μm pore size. In the co-culture system, we co-cultured ECs with either UBE2CP3 overexpressing or UBE2CP3 knockdown HCC cells. The results showed that in the co-culture system, overexpressing UBE2CP3 in HCC cells enhances EC proliferation, migration and tube formation abilities, and knocking down UBE2CP3 induced the opposite results. In addition, we investigated the biological function of UBE2CP3 in angiogenesis by chick embryo chorioallantoic membrane (CAM) angiogenesis and nude mouse tumorigenicity assay, which showed that UBE2CP3 up-regulates EV density in vivo. Our study suggests that UBE2CP3 may play an important role in the angiogenesis of HCC.

## Methods

### Antibodies, inhibitor and neutralizing antibody

Antibodies against ERK (Proteintech, USA), p-ERK (Cell Signalling Technology, Beverly, MA, USA), P70S6K (Cell Signalling Technology), p-P70S6K (Cell Signalling Technology), HIF-1α (Abcam, Cambridge, UK), VEGFA (ABclonal, Wuhan, China) were used in Western blotting (WB). CD31 (Abcam) antibody was used for IHC. PD98059(Selleck Chemicals, Houston, USA) were used to inhibit p-ERK. Neutralizing antibody to VEGFA165 was used to block the effects of VEGFA in cell supernatant (R&D systems, Minneapolis, USA).

### Patient samples and inclusion criteria

Two independent cohorts comprising a total of 94 HCC patients were enrolled in this study. In cohort 1, formalin-fixed, paraffin-embedded tissues from 48 HCC patients were used for IHC and ISH. All patients in cohort 1 were followed up for 5 years after surgery; the detailed clinical information for these patients is shown in Table 1. In cohort 2, fresh HCC samples and corresponding para-tumor tissues were obtained from 46 HCC patients who were undergoing routine surgery at Nangfang Hospital, Southern Medical University (Guangzhou, China); the clinical information for these patients is shown in Table 2. All patients provided written informed consent, and the research protocol was approved by the Nangfang Hospital (Guangzhou, China) Ethics Committee.

**Table 1:** Correlation among UBE2CP3, EV density and clinicopathological parameters of HCC patients in cohort1

	UBE2CP3			EV		
	Low(n)	High(n)	*P*	Low(n)	High(n)	*P*
All cases	26	22		31	17	
Age(year)						
>=55	16	13	0.863	17	12	0.286
<55	10	9		14	5	
Sex						
Male	21	21	0.274	28	14	0.732
Female	5	1		3	3	
Edmondson grade						
I~II	20	13	0.184	21	12	0.839
III~IV	6	9		10	5	
Tumor invasion^a^						
T1~T2	15	7	0.037*	16	6	0.193
T3~T4	9	15		13	11	
Cirrhosis						
Without	17	11	0.281	18	10	0.959
With	9	11		13	7	
Tumor number						
Single	26	14	0.003**	29	11	0.031*
Multiple	0	8		2	6	
Tumor size,*cm*						
<=10	20	12	0.101	24	8	0.033*
>10	6	10		7	9	
Mortality						
Survive	15	5	0.014*	16	4	0.059
Die	11	17		15	13	

Abbreviations: *EV* endothelial vessel, *UBE2CP3* ubiquitin conjugating enzyme E2 C pseudogene 3. *P < 0.05, ** *P* < 0.01

^a^Remarks: 2 records of tumor invasion were missing

**Table 2:** Correlation among UBE2CP3, CD31 mRNA and clinicopathological parameters of HCC patients in cohort 2

	UBE2CP3			CD31 mRNA		
	Low(n)	High(n)	*P*	Low(n)	High(n)	*P*
All cases	22	24		29	17	
Age(year)						
>=55	13	14	0.958	17	10	0.989
<55	9	10		12	7	
Sex						
Male	16	20	0.384	21	15	0.376
Female	6	4		8	2	
Edmondson grade						
I~II	14	12	0.351	15	11	0.391
III~IV	8	12		14	6	
Tumor invasion						
T1~T2	14	7	0.019*	16	5	0.090
T3~T4	8	17		13	12	
Cirrhosis						
Without	13	12	0.536	18	7	0.170
With	9	12		11	10	
Tumor number						
Single	21	16	0.014*	27	10	0.015*
Multiple	1	8		2	7	
Tumor size,*cm*						
<=10	15	11	0.127	20	6	0.026*
>10	7	13		9	11	

The median expression level was used as the cut off. Low expression of UBE2CP3 in 22 patients was classified as values below the 50^th^ percentile. High UBE2CP3 expression in 24 patients was classified as values at or above the 50^th^ percentile.

Abbreviations: *EV* endothelial vessel, *UBE2CP3* ubiquitin conjugating enzyme E2 C pseudogene 3. *P < 0.05, ** *P* < 0.01

### IHC and ISH

**Fig. 1:**
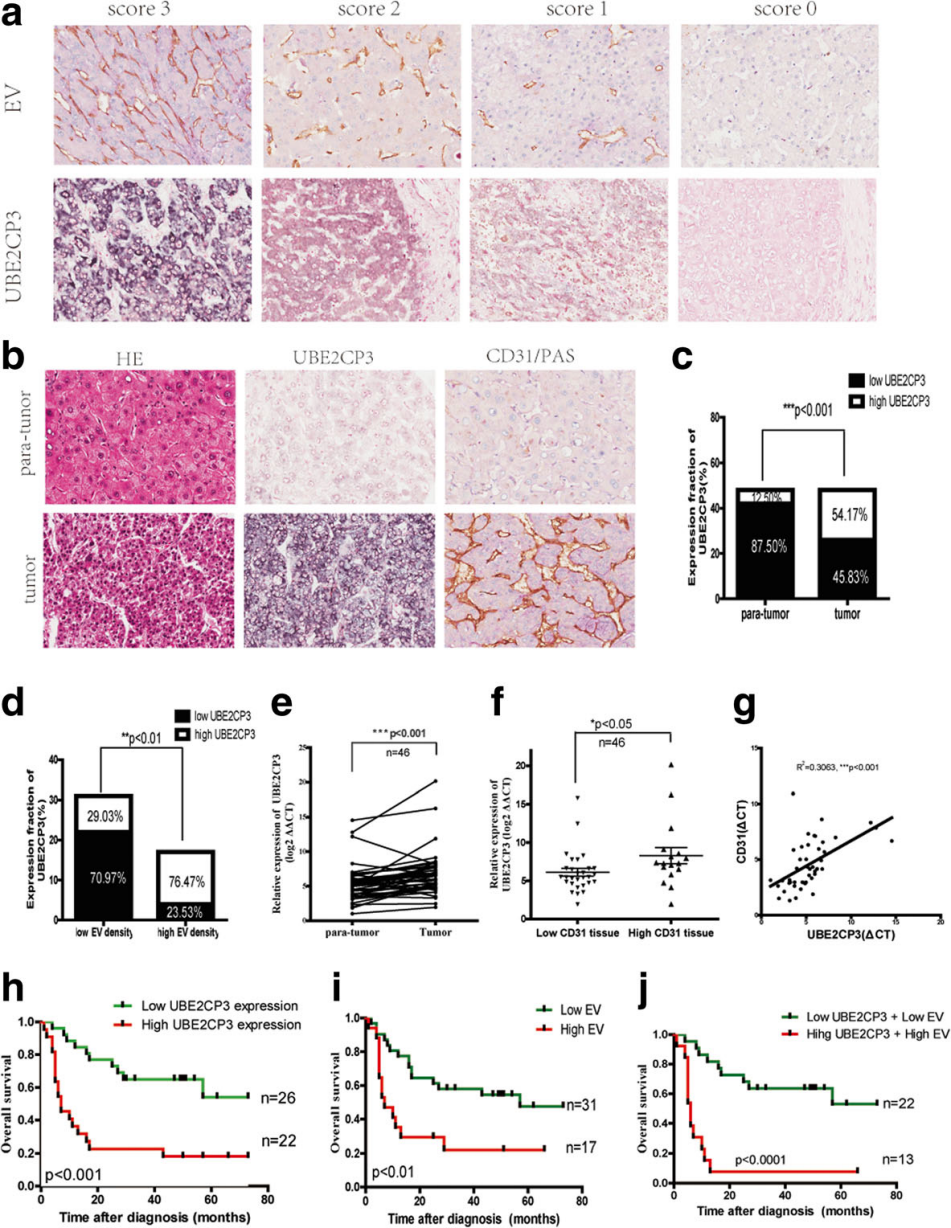
UBE2CP3 is frequently up-regulated in HCC tissues and in tissues with high EV density and is associated with HCC patient prognosis. **a** Representative images of different intensities of UBE2CP3 ISH staining and of CD31/PAS double-staining for EV (CD31+). **b**, **c**, **d** Serial sections were stained with haematoxylin and eosin for H&E. ISH was used to examine UBE2CP3 expression and orientation. CD31/PAS double-staining was used to determine the expression of EV density. The results showed that UBE2CP3 was upregulated. **e**, **f** qRT-PCR analysis showed that UBE2CP3 expression was higher in HCC tissues than in para-tumor tissues (**e**) and was upregulated in HCC tissues with high CD31 mRNA expression (**f**). **g** The correlation between UBE2CP3 expression level and CD31 mRNA level in 46 HCC tissues. **h**, **i** Patients with high UBE2CP3 expression (**h**) and EV density (**i**) had a shorter overall survival time (OS) (*P*=0.0040, and *P*=0.0069, respectively). **j** Log-rank (Mantel-Cox) tests showed that when grouped by both UBE2CP3 and EV expression, HCC patients with high UBE2CP3 expression and EV density had a worse OS (*P*=0.0003)

IHC assays were performed with anti-VEGFA antibody and CD31/periodic acid-Schiff (PAS) double-staining. The ISH probe used for detecting UBE2CP3-labelled digoxin was designed and synthesized by Exiqon (Shanghai, Chia). The probe sequence is listed in Additional file 1: Table S1. ISH was performed using an ISH Kit (Boster Bio-Engineering Company, Wuhan, China) in accordance with the manufacturer’s instructions. The scoring for staining intensity was as follows: 0 (negative staining), 1 (weak), 2 (medium), 3 (strong) (Fig. 1c). The score of staining extent was as follows: 0 (<10%), 1 (11%-25%), 2 (26%-50%), 3 (51%-75%), and 4 (76%-100%). The final UBE2CP3 expression score was calculated as the intensity score × the extent score, and it ranged from 0 to 12. Sections with a total score of 6 or higher were considered as the high expression group, and those with a score less than 6 were categorized as the low expression group. The IHC and ISH scores were evaluated by two pathologists in a blinded manner. When their opinions were inconsistence, a third pathologist who was also blinded to the patient information was asked to give the final score.

### Cell lines and cultures

Human HCC cell lines (HepG2 and SMMC-7721) and HUVECs were purchased from the Cell Bank of Type Culture Collection (CBTCC, Chinese Academy of Sciences, Shanghai, China). Cells were grown in Dulbecco’s modified Eagle’s medium (DMEM, Gibco, Gaithersburg, MD, USA) containing 10% foetal bovine serum (FBS) in a humidified incubator at 37 °C with 5% CO_2_.

### Construction of stable cell lines

To obtain cell lines stably overexpressing UBE2CP3, HepG2 and SMMC7721 cells were infected with Lv-UBE2CP3 and Lv-control viruses (Land, Guangzhou, China). To study the knockdown effects of UBE2CP3 in vitro, HepG2 and SMMC7721 cells were transfected with shRNA-UBE2CP3 (Sh-UBE2CP3) or control (Sh-control) viruses (Obio Technology, Shanghai, China). The infection efficiency was confirmed by qRT-PCR.

### Cell co-culture

Co-culture inserts (0.4-μm pores; Corning, USA) were placed into 6-well culture plates. HUVECs were added to the lower culture wells (2 × 10 ^[5]^ cells per well), and HepG2 and SMMC7721 cells (2 × 10 ^[5]^ cells per well) were placed in the inserts and cultured in DMEM supplemented with 5% FBS for 48 h; then, the HUVECs were harvested for biological function studies.

### Cell culture supernatant concentration determination

The supernatants from either UBE2CP3 overexpressing or UBE2CP3 knockdown HepG2 and SMMC-7721 cells were collected. Cell culture supernatants were centrifuged at 1,000 g for 10 min to remove the cells and cell debris; then, the supernatants were added to an Amicon Ultra filter device (Millipore, Billerica, MA, USA) and were centrifuged at 4,000 g for 30 min to obtain the concentrated, protein-containing liquid.

### Enzyme-linked immunoassay (ELISA)

VEGFA in the medium was measured by using the Quantikine human VEGF ELISA kit (R&D Systems, Minneapolis, USA) according to manufacturer’s instruction.

### RNA isolation and RT-PCR

Total RNA from cultured cells or human tissue was extracted using TRlzol reagent (Takara, Dalian, China) according to the manufacturer’s instructions. The mRNA levels were measured by qRT-PCR using SYBR Green PCR Master Mix (Takara) by with an ABI 7500 Fast Real-Time PCR system. The expression of β-actin was used as the internal control for analysing the mRNA level of VEGFA. The expression of U6 was used as the internal control for analysing lncRNA UBE2CP3 expression in tissues and cells. Comparative quantification was determined using the 2^-ΔΔ^CT method. All samples were measured with at least three independent experiments, and the results are expressed as the means ± SD for comparative analysis. The primers used are listed in Additional file 1: Table S1.

### Western blot analysis

Proteins from HepG2 and SMMC7721 cells and from the concentrated cell culture supernatant were extracted with protein extraction kits (Keygen Biotech, Jiangsu, China) containing protease and phosphatase inhibitors. The protein concentration was quantified using a bicinchoninic acid (BCA) protein assay kit (Keygen Biotech). Then, proteins were separated on a 10% sodium dodecyl sulfate-polyacrylamide gel and transferred onto polyvinylidene difluoride membranes. The membranes were blocked with 5% BSA for 1 h at room temperature and incubated with primary antibodies overnight at 4°C. Next, the membranes were washed with TBST three times and incubated with goat anti-rabbit secondary antibodies for 1 h at room temperature; the protein bands were visualized using a chemiluminescence detection system (ECL Plus Western Blot Detection System; Amersham Biosciences, Foster City, CA) according to the manufacturer's protocol. Quantification was completed by Image J software.

### 5-Ethynyl-2′-deoxyuridine (EdU) incorporation assay

Following the manufacturer’s instructions, we used the Cell-Light EdU imaging detection kit (RiboBio, Guangzhou, China) for EdU incorporation assays. HUVECs were harvested from the co-culture system, and after being washed twice with PBS, the cells were seeded into 96-well plates at a density of 5000 cells/well. Six h later, we added EdU labelling medium and incubated the cells for approximately 60 min after they adhered to the plate surface; then, the cells were fixed with 4% formaldehyde for 15 min, treated with 0.5% Triton X-100 for 20 min and exposed to Apollo reaction cocktail for 30 min. Next, in each well, the cellular DNA was stained with Hoechst 33342 (5 up/mL) for 30 min. Finally, we visualized the results under a fluorescence microscope.

### Cell cycle analysis

Cells were harvested from the co-culture system; after being washed twice with PBS, the cells were fixed in 1 ml of 70% ice-cold ethanol and were stored overnight at 4°C. Subsequently, the cells were stained with propidium iodide supplemented with RNaseA (Keygen Biotech) for 30 min at 37°C. The DNA content of the labelled cells was analysed using FACS flow cytometry (BD Biosciences Inc., Franklin Lakes, NJ, USA). Each experiment was performed three times.

### Tube formation assay

For tube formation assay, 50 μl of Matrigel (BD Biosciences, San Jose, CA, USA) was added to each well of a 96-well plate. After polymerizing at 37°C for 30 min, HUVECs were harvested from the co-culture system and were seeded onto the Matrigel at the density of 30,000 cells per well. After incubation for 6 h in a humidified incubator at 37°C with 5% CO_2_, tubules were visualized under an IX71 inverted microscope.

### Cell migration assay

All cell migration assays in vitro were performed using Transwell chambers (8-μm pore size; Corning) in 24-well plates. After being suspended in DMEM with 10% FBS, HCC cells with UBE2CP3 overexpressed or knocked down along with the corresponding control groups were seeded at 50,000 cells per well into the bottom chamber. These HCC cells were cultured in the lower chamber for 24 h; then, in the upper chamber, we added a total of 30,000 HUVECs that had been harvested from the co-culture system. After an18-h incubation, the cells in the chamber were fixed with methanol, followed by staining with 0.1% crystal violet. Cells on the upper side of the Transwell membrane were wiped off with a cotton swab, and cells on the underside were photographed under a microscope and quantified.

### Wound healing assay

HUVECs were harvested from the co-culture system, and each group was washed, suspended in DMEM, and seeded at a density of 500,000 cells per well in 6-well culture plates. After culturing for 24 h and reaching 100% confluence, wounds were made with a 200-μl plastic pipette tip. After being photographed at h 0, co-culture inserts with HepG2 or SMMC-7721 cells (at a density of 200,000 cells per insert) were placed into 6-well culture plates. After incubating in a humidified incubator at 37°C with 5% CO_2_ for 18 h, the wound width was measured and recorded using an IX71 inverted microscope. Lastly, the results from three wound healing assays were calculated and analysed.

### Xenograft tumors in vivo

Our animal investigations were performed in accordance with the institutional guidelines and were approved by the Animal Experimental Committee of Nanfang Hospital. Four-week-old male BALB/c nude mice were purchased from the Guangdong Experimental Animal Center of the Chinese Academy of Sciences and were bred and maintained in specific pathogen-free conditions. Cells from each group were resuspended in serum-free DMEM at a density of 50,000,000 cells per ml and then 0.1 ml of the suspension was injected into the back of the nude mice. Tumor tissues were obtained 5 weeks later for IHC.

### Angiogenesis assay in chick chorioallantoic membranes (CAM)

After being cultured at 37 °C for 7 days, a window was opened on the shell of fertilized eggs to expose the CAM and then was covered with a filter paper disc (0.5 cm in diameter) containing HepG2 cells (18,000/disc) or PBS on the surface. Next, tape was used to cover the window for further incubation. Two days later, the CAM was fixed in 3.7% formaldehyde, and we visualized the results under a stereoscope.

### Statistical analysis

Data were shown as the means±SD of at least three independent experiments. Statistical analysis was performed using SPSS 13.0 software (SPSS Inc., Chicago, IL, USA) and GraphPad Prism (GraphPad Software, Inc., La Jolla, CA, USA). The chi-Square test was used to examine the relationship between EV density, UBE2CP3 expression and clinicopathological characteristics. Differences between experimental groups were assessed by Student’s t-test or one-way ANOVA. Kaplan-Meier and log-rank tests were used to analyse survival time. Statistical significance was set at *P < 0.05, **P < 0.01, ***P < 0.001. *P* < 0.05 was considered statistically significant.

## Results

### The expression of UBE2CP3 was frequently up-regulated in HCC tissues, especially in high EV density tissues, and UBE2CP3 expression combined with EV density was associated with HCC patient prognosis

To identify the relationship between UBE2CP3 expression and HCC angiogenesis, we examined UBE2CP3 expression levels by ISH and qRT-PCR. We also concomitantly used CD31/PAS double-staining to measure EV density and used qRT-PCR to measure the CD31 mRNA level. The representative images are shown in Fig. 1a. UBE2CP3 expression was higher in HCC tissues than in para-tumor tissues (Fig. 1b, c and e) and was up-regulated in high EV density tissues compared with its level in low EV density tissues (Fig. 1b, d and f). Moreover, the levels of UBE2CP3 were positively correlated with CD31 mRNA levels (R ^[2]^=0.3063, *P*<0.001 Fig. 1g). Kaplan-Meier and log–rank test analyses revealed that the high level of UBE2CP3 (Fig. 1h) and high EV density (Fig. 1i) were associated with reduced overall survival time (OS). When grouped by both UBE2CP3 expression and EV density, HCC patients in the high category had poor OS (Fig. 1j). The correlations among UBE2CP3 expression levels, EV density and the clinicopathological parameters of the HCC patients in cohort 1 are shown in Table 1. The correlations among UBE2CP3 expression levels, CD31 mRNA level and the clinicopathological parameters of the HCC patients in cohort 2 are shown in Table 2. Chi-square test revealed that both the levels of UBE2CP3 (*P*=0.003,Table 1; *P*=0.014, Table 2) and EV density (*P*=0.031,Table 1; *P*=0.015, Table 2) were significantly correlated with tumor numbers. Furthermore, the levels of UBE2CP3 were positively correlated with mortality (*P*=0.014,Table 1) and tumor invasion (*P*=0.037, Table 1; *P*=0.019, Table 2); and EV density was significantly correlated with tumor size (*P*=0.033, Table 1; *P*=0.026, Table 2).

### UBE2CP3 in HCC cells indirectly affected EC proliferation, migration, and tube formation in the co-culture system

**Fig. 2:**
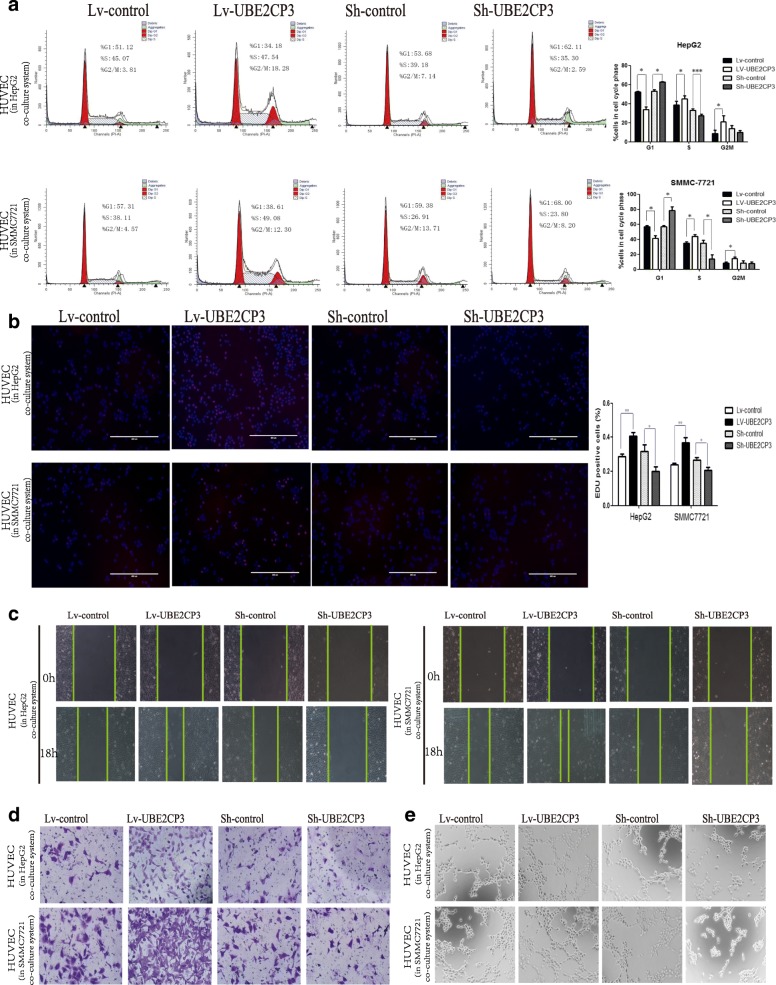
UBE2CP3 enhanced the crosstalk between HCC cells and HUVECs, and promoted cell cycle progression, migration and tube formation in HUVECs which co-cultured with HepG2/SMMC-7721 cells. **a** Analysis of cell cycle progression in HUVECs after co-culturing with UBE2CP3 overexpressing HepG2/SMMC-7721 cells or with UBE2CP3 knockdown HepG2/SMMC-7721 cells. **b** HepG2/SMMC-7721 cells with increased UBE2CP3 expression were seeded onto 24-well plates, HUVECs were seeded into the co-culture inserts, and cell proliferation was examined by EdU immunofluorescence staining. The effect of UBE2CP3 knockdown on HepG2/SMMC-7721 cell proliferation was also measured by EdU immunofluorescence staining. The graph on the right shows the percentage of EdU-positive nuclei. **c**, **d** UBE2CP3 promoted tumor-induced HUVEC migration according to wound healing (**c**) and Transwell migration assays (**d**). **e** UBE2CP3 promoted tumor-induced HUVEC angiogenesis according to tube formation assays. The results show the means ± SD from at least three separate experiments. *P < 0.05, **P < 0.01, ***P < 0.001

Our ISH and IHC results indicated that UBE2CP3 may participate in the HCC angiogenic process. The indirect effects of UBE2CP3 on ECs were studied by establishing a co-culture system using Transwell chambers with a 0.4-μm pore size. HCC cells that had been transfected with Lv-UBE2CP3 or Sh-UBE2CP3 virus (the infection efficiencies are shown in Additional file 2: Figure S1A) were seeded into the upper chamber, and ECs were seeded into the lower chamber. HCC cells and ECs from the co-culture system were separated, but the cytokines and growth factors were able to communicate with each other (Additional file 2: Figure S1B). In the co-culture system, ECs were exposed to HCC cells for 48 h. Following this exposure, ECs were removed from the co-culture system for further studies. Since angiogenesis involves EC proliferation, migration and tube formation, we analysed the cell cycle and performed EdU, Transwell, wound healing and tube formation assays to gain insight into the role of UBE2CP3 in cell proliferation, migration and tube formation. We observed that compared with the control group, EC cells co-cultured with HCC cells stably overexpressing UBE2CP3 had significantly enhanced proliferation (Fig. 2a, b), migration (Fig. 2c, d) and tube formation potential (Fig. 2e). Conversely, ECs co-cultured with the UBE2CP3-knockdown HCC cells had decreased proliferation (Fig. 2a, b), migration (Fig. 2c, d) and tube formation abilities (Fig. 2e). Our results suggested that the dysregulation of UBE2CP3 in HCC cells may play an indirect effect on ECs proliferation, migration and tube formation in vitro.

### UBE2CP3 in HCC cells promoted EC proliferation, migration, and tube formation by enhancing the secretion of VEGFA into the supernatant via activation of the ERK/HIF-1α signalling pathway

**Fig. 3:**
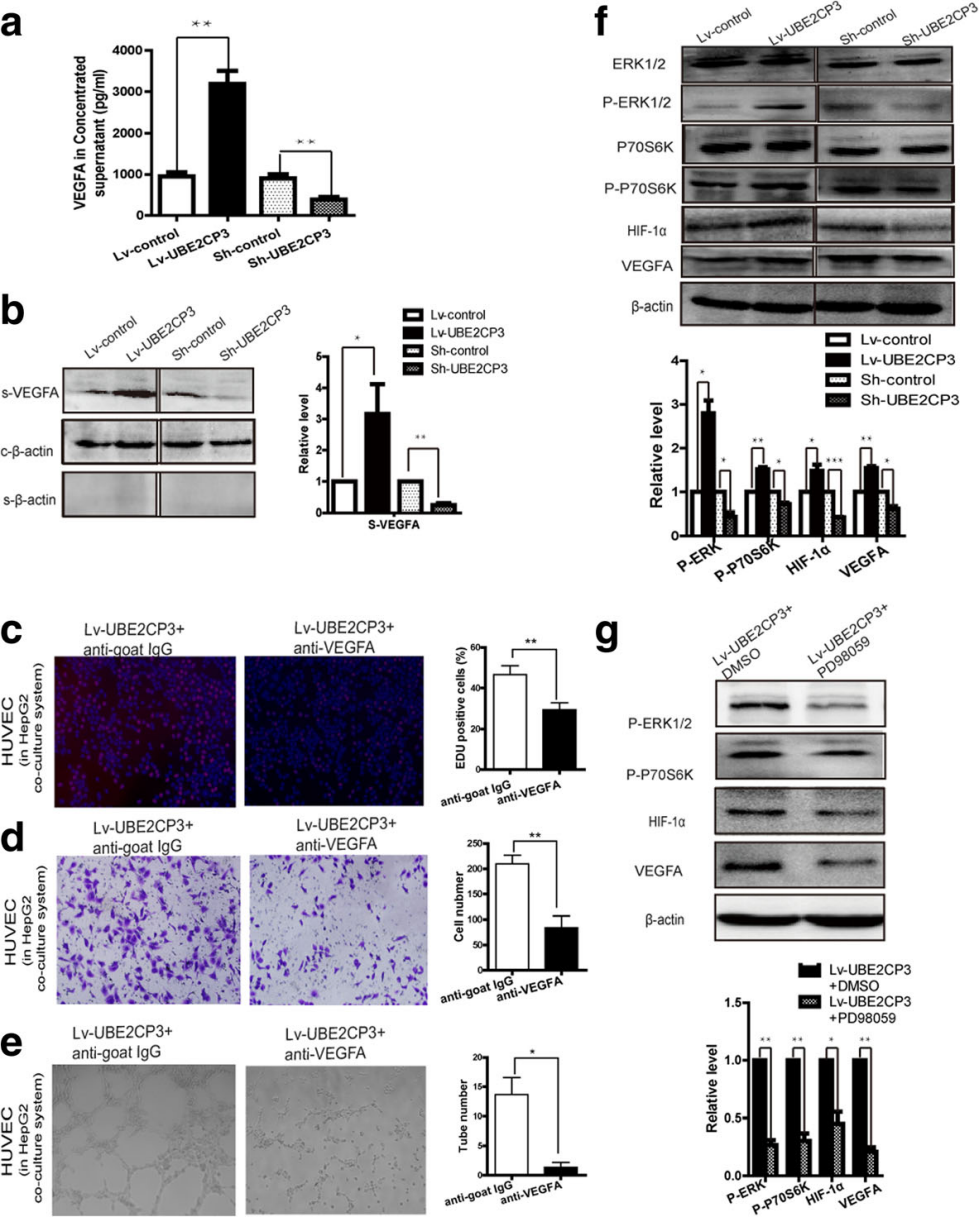
UBE2CP3 in HCC cells promoted EC proliferation, migration, and tube formation by enhancing the secretion of VEGFA into the supernatant via activation of the ERK/HIF-1α signalling pathway. **a**, **b** The levels of VEGFA in HepG2 concentrated supernatant were analysed by ELSIA (**a**) and western blot (**b**). For western blot, β-actin served as the internal control, β-Actin in the cell supernatant served as the quantity control. **c**, **d**, **e** Using VEGFA neutralizing antibody markedly reduce the effects of UBE2CP3 in HCC cells on EC proliferation (**c**), migration (**d**) and tube formation (**e**) in the co-culture system, IgG antibodies were used for negative control. **f** The levels of ERK1/2, p-ERK, p70S6K, p-p70S6K, HIF-1α, and VEGFA were examined by Western blot analysis in HepG2 cells overexpressing UBE2CP3 and in HepG2 cells with UBE2CP3 expression silenced. **g** Treatment with p-ERK inhibitor (PD98059) markedly reduce the levels of p-ERK, p-p70S6K, HIF-1α and VEGFA in UBE2CP3 overexpressing HepG2 cells. The data are expressed as the means ± SD. *P < 0.05, **P < 0.01, ***P < 0.001

ECs co-cultured with UBE2CP3-dysregulated HCC cells had alterations in cell proliferation, migration and tube formation abilities. We hypothesized that cytokines and/or growth factors may participate in this process. As shown in Additional file 2: Figure S1C, 12 kinds of common angiogenic factors were detected using qRT-PCR, the mRNA levels of VEGFA (up-regulated fold=4.04, *P*=0.008), Ang2(up-regulated fold=1.82, *P*=0.031) were increased in UBE2CP3 overexpressing HepG2 cells and the mRNA levels of VEGFA(down-regulated folds=0.31, *P*=0.008), Ang2(down-regulated folds=0.63, *P*=0.037), and PDGFC(down-regulated folds=0.49, *P*=0.038) were decreased in UBE2CP3 knocking down HepG2 cells. Since VEGFA is one of the most important growth factors in angiogenesis and is the most obvious changing cytokine in UBE2CP3 overexpressing or knocking down HepG2 cells, we decided to investigate the role and the underlying mechanism of VEGFA in UBE2CP3 inducing angiogenesis in HCC. The VEGFA levels in the Lv-control, Lv-UBE2CP3, sh-control, and sh-UBE2CP3 cell supernatants from the co-culture system after concentrated by an Amicon Ultra filter device were determined by WB and ELISA assay. As expected, a higher level of VEGFA was observed in the UBE2CP3 overexpressing HCC cell supernatant than in the supernatant of the control cells. In contrast, in the co-culture system, knocking down UBE2CP3 induced a decrease in the supernatant VEGFA level (Fig. 3a, b). To investigate whether VEGFA take part in UBE2CP3 inducing EC proliferation, migration and tube formation, VEGFA neutralizing antibodies were used to block the VEGFA effects in the co-culture system. When adding VEGFA neutralizing antibodies, the indirect effects of UBE2CP3 on EC proliferation, migration, and tube formation in the co-culture system were significantly reduced. (Fig. 3c, d, e). Meanwhile, the WB assays with HCC cell protein showed higher levels of phosphor -ERK (p-ERK), phosphor-p70S6K (p-p70S6K), HIF-1α, and VEGFA in the UBE2CP3 overexpressing cells than in the control cells. In contrast, knocking down the expression of UBE2CP3 reduced the levels of p-ERK, p-p70S6K, HIF-1α, and VEGFA (Fig. 3b, c) compared with their expression in the control cells (Fig. 3f). After treated with 100μM PD98059 for 48h, the level of p-ERK was significantly reduced, and the levels of p-p70S6K, HIF-1α, and VEGFA can also be reduced by the inhibitor (Fig. 3g).

### UBE2CP3 promoted angiogenesis in vivo

**Fig. 4:**
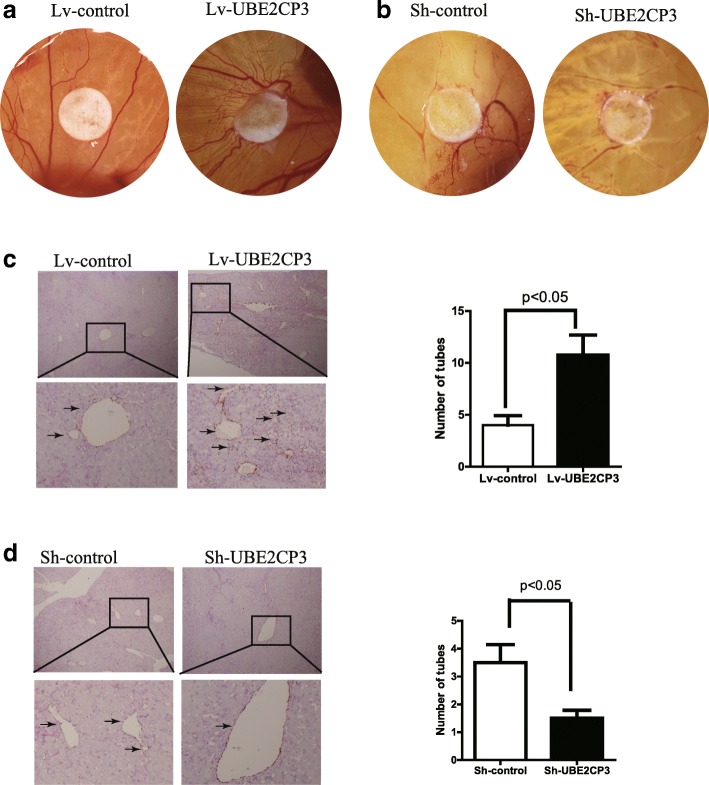
UBE2CP3 promoted angiogenesis in vivo. **a** CAM angiogenesis assays showed that when injected with HCC cells overexpressing UBE2CP3, the density of new chick embryo vessels increased. **b** Chick embryos injected with UBE2CP3 knockdown cells had decreased new vessel density. **c** CD31/PAS double-staining results showed that mice injected with cells overexpressing UBE2CP3 had higher EV density in the tumor tissue than those injected with Lv-control cells. **d** Mice injected with UBE2CP3 knockdown cells had lower EV density in the tumor tissue than those injected with the sh-control cells

To determine the effects of UBE2CP3 on HCC angiogenesis, we injected HepG2 cells that were stably transfected with Lv-UBE2CP3 or Lv-control into chick chorioallantoic membranes (CAM). Chick embryos injected with cells overexpressing UBE2CP3 had an increase in new vessel density (Fig. 4a). In contrast, when chick embryos were injected with cells with inhibited UBE2CP3 expression, a decrease in new vessel density was observed (Fig. 4b). Consistent with these results, mice injected with cells overexpressing UBE2CP3 had a higher EV density in the tumor tissue than the mice injected with Lv-control cells (Fig. 4c). Mice injected with cells knocking down UBE2CP3 had a lower EV density in the tumor tissue than the mice injected with sh-control cells (Fig. 4d).

## Discussion

Angiogenesis is one hallmark of cancer [17], when growing to a certain size, a tumor mass requires angiogenesis to maintain its nutritional supply for continued development. In most instances, tumor cells secrete angiogenic substances and initiate angiogenesis process [18]. Tumor angiogenesis requires interactions among tumor cells, ECs and mesenchymal cells through growth factors or cytokines and their corresponding receptors. Recent studies have suggested that lncRNAs can modulate the process of angiogenesis by regulating the expression level of angiogenic molecules and the functions in ECs [19, 20]. LncRNAs are a type of RNA that are longer than 200 nucleotides and do not have protein-coding capacity. An increased amount of evidence suggests that lncRNAs play an important role in regulating a wide variety of biological processes related to hepatocarcinogenesis, including cell survival, apoptosis, metastasis, and angiogenesis [9, 21, 22]. Although several angiogenesis-associated lncRNAs have been identified, the function and clinical significance of most angiogenic lncRNAs in HCC angiogenesis remain largely unknown. In addition, the effects of lncRNAs on the regulation of the interactions between tumor cells and ECs have not been identified.

In this study, we found that the expression of UBE2CP3 was up-regulated in HCC tissues compared to the levels in para-tumor tissues, and compared to its expression in tissues with low EV density, UBE2CP3 expression was up-regulated in tissues with high EV density. The clinical results showed that UBE2CP3 expression and EV density are both significantly correlated with metastasis (i.e., positively correlated with tumor number) and poor prognosis in HCC patients. When categorized according to both UBE2CP3 expression and EV density, HCC patients in the high category presented with poor OS. These results suggested that a high level of UBE2CP3 may be closely related to HCC angiogenesis and that UBE2CP3 might serve as a valuable therapeutic target in HCC. Consistent with our results, Cao SW et al [15] found that UBE2CP3 plays an important role in promoting HCC metastasis by inducing EMT. An increasing amount of evidence suggests that EMT is closely related to angiogenesis [23–25]; angiogenesis is promoted by EMT-induced VEGFA in human breast tumors [16] and is enhanced by the EMT-mediated increase in the secretion of factors known to enhance angiogenesis (e.g., TGF-β, CSF-1, NGF, VGF, ADAM9 and ADAM17) into the extracellular microenvironment [26]. Considering the above findings, there is a strong internal relationship between angiogenesis and EMT; therefore, the role of UBE2CP3 in HCC angiogenesis is clearly indicated.

Since the EMT process has been suggested to be closely related to the tumor extracellular microenvironment, we hypothesized that UBE2CP3 may play an important role in the tumor extracellular microenvironment. Thus, in this study, we performed gain- and loss-of-function experiments in HCC cells. We first investigated the indirect role of UBE2CP3 on EC proliferation, migration, and tube formation by constructing a co-culture system with HCC cells and ECs by using a Transwell chamber with a 0.4-μm pore size; in the co-culture system, HCC cells and ECs were separated, but cytokines and growth factors were still able to communicate with each other. We demonstrated that HCC cells stably overexpressing UBE2CP3 in the co-culture system promoted EC proliferation, migration and tube formation by enhancing the secretion of VEGFA into the supernatant. It has been well defined that VEGFA is one of the most important growth factors for angiogenesis [27]. VEGFA can activate relevant angiogenic signalling pathways such as those of ERK, phosphoinositide 3 kinase (PI3K)/Akt [28], phospholipase Cγ (PLCγ) [29, 30], Src, focal adhesion kinase (FAK) [31], p38 mitogen-activated protein kinase (MAPK) [32], the Rho family [33], GTPases [34] and endothelial nitric oxide (NO) [35]. These signalling pathways eventually alter EC proliferation, migration, invasion and tube formation capacity and vascular permeability. In summary, the secretion of VEGFA into the HCC cell extracellular microenvironment might be an important mediator of UBE2CP3-regulated angiogenesis. However, which signalling pathways are activated in ECs by the ectopic expression of UBE2CP3 in HCC cells requires further investigation.

An increasing amount of evidence demonstrates that hypoxia inducible factor-1α (HIF-1α) is the main upstream inducer of VEGFA, which plays a key role in tumor angiogenesis [36, 37]. Recent studies have characterized a series of HIF-1α related pathways, such as the ERK [38], TNF-α [39], phosphatidylinositol-3-kinase (PI3K), mammalian homologue target of rapamycin (mTOR), and AKT [40, 41] pathways. Here, we found that UBE2CP3 up-regulated the levels of p-ERK, phosphor-p70S6K (p-p70S6K), HIF-1α, and VEGFA in HCC cells. In contrast, knocking down the expression of UBE2CP3 reduced the levels of p-ERK, p-p70S6K, HIF-1α and VEGFA. ERK signalling has been reported to not only participate in various tumor transitions for cell survival and invasion [42, 43] but also modulate the transcription factors for tumor EMT and angiogenesis [44, 45]. P70S6K is one of the ERK pathway effectors, and an increasing amount of evidence demonstrates that ERK/p70S6K signalling participates in several pathological processes, such as cell drug-resistance and angiogenesis [46, 47]. In addition, p70S6K modulates the expression and activation of HIF-1α [48, 49]. Our results demonstrated that the ERK/p70S6K/HIF-1α signalling pathway may be partially responsible for the UBE2CP3-induced accumulation of VEGFA and leads to tumor cell-mediated angiogenesis.

Although we confirmed that UBE2CP3 may act as an oncogene to promote the secretion of VEGFA from HCC cells into the tumor microenvironment by activating the ERK/p70S6K/HIF-1α pathway and enhance tumor cell-induced angiogenesis, the underlying mechanism of how ECs respond to UBE2CP3 dysregulation in HCC cells is still unclear. Moreover, because our study was based on the co-culture system, adding ERK signalling inhibitors in the co-culture system will not only inhibited the ERK pathway in HCC cells but also reduce the activity of ERK in endothelial cells, a more appropriate experimental design is needed to study the function of UBE2CP3 when ERK signalling is inhibited. Additionally, the molecules functioning directly downstream of UBE2CP3 remain unknown; further research, such as pull-down assays, is needed to determine the direct target molecules of UBE2CP3.

## Conclusions

**Fig. 5:**
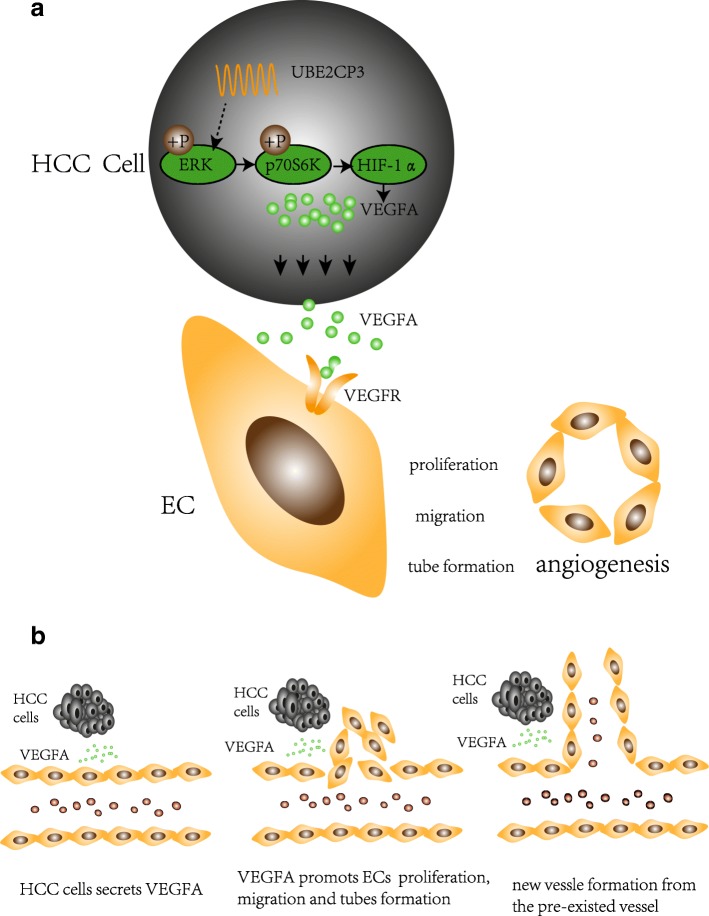
Diagrammatic sketches of UBE2CP3 promotion of tumor cell-induced angiogenesis. **a** UBE2CP3 promoted the secretion of VEGFA in HCC cells by activating ERK/HIF-1α signalling. **b** The HCC-induced process of forming new vessels from pre-existing ones

As summarized in Fig. 5, this study demonstrates that lncRNA UBE2CP3 promotes angiogenesis indirectly. Specifically, UBE2CP3 promotes HCC cell secretion of VEGFA into the tumor microenvironment by activating the ERK/p70S6K/HIF-1α pathway; this VEGFA alters EC proliferation, migration and tube formation capacities. Modulating tumor angiogenesis by inhibiting UBE2CP3 expression may be a potential strategy for HCC prevention and treatment. Our results suggest that in HCC, UBE2CP3 indirectly enhances tumor cell-induced angiogenesis. UBE2CP3 is a potential oncogene that participates in HCC tumorigenicity by facilitating angiogenesis.

## Additional files

Additional file 1: **Table S1.** Sequences of primers and probe sequences used in this study. (DOC 14 kb)

Additional file 2: **Figure S1.** (**A**) The infection efficiencies of lncRNA UBE2CP3 in HepG2 and SMMC-7721 were screened by qRT-PCR. (**B**) The schematic diagram for the co-culture system. (**C**) 12 kinds of common angiogenic factors were detected by qRT-PCR.. (TIF 24 kb)

## Abbreviations

CAM: chick embryo chorioallantoic membrane
EMT: epithelial–mesenchymal transition
EV: endothelial vessel
HCC: hepatocellular carcinoma
HUVEC: human umbilical vein endothelial cell
IHC: immunohistochemistry
ISH: in situ hybridization
lncRNA: long non-coding RNA
ORF: open reading frame
OS: overall survival time
qRT-PCR: quantitative real-time polymerase chain reaction
UBE2CP3: ubiquitin conjugating enzyme E2C pseudogene 3
