# Supplementary material for: Long non-coding RNA UBE2CP3 enhances HCC cell secretion of VEGFA and promotes angiogenesis by activating ERK1/2/HIF-1α/VEGFA signalling in hepatocellular carcinoma
Source: J Exp Clin Cancer Res. 2018 Jun 4;37:113. doi: 10.1186/s13046-018-0727-1 (PMC5987644; doi:10.1186/s13046-018-0727-1)
Supplement: Supplementary file 1 — Table S1. Sequences of primers and probe sequences used in this study. (DOC 14 kb) [file 13046_2018_727_MOESM1_ESM.doc]

Additional file1:

Table S1 Sequences of primers and probe sequences used in this study

| Name |  | Sequences |
| --- | --- | --- |
| qRT-PCR primers |  |  |
| lncRNA UBE2CP3 | sense | AAGTGGTCTGCCCTGTATGATG |
|  | antisense | GAGCTATCAATGTTGGGTTTGC |
| U6 | sense | CTCGCTTCGGCAGCACA |
|  | antisense | AACGCTTCACGAATTTGCGT |
| β-actin | sense | TGGCACCCAGCACAATGAA |
|  | antisense | CTAAGTCATAGTCCGCCTAGAAGCA |
| CD31 | sense | AACAGTGTTGACATGAAGAGCC |
|  | antisense | TGTAAAACAGCACGTCATCCTT |
| VEGFA | sense | AGGGCAGAATCATCACGAAGT |
|  | antisense | AGGGTCTCGATTGGATGGCA |
| Ang2 | sense | AACTTTCGGAAGAGCATGGAC |
|  | antisense | CGAGTCATCGTATTCGAGCGG |
| Ang1 | sense | AGCGCCGAAGTCCAGAAAAC |
|  | antisense | TACTCTCACGACAGTTGCCAT |
| HGF | sense | GCTATCGGGGTAAAGACCTACA |
|  | antisense | CGTAGCGTACCTCTGGATTGC |
| bFGF | sense | AGAAGAGCGACCCTCACATCA |
|  | antisense | CGGTTAGCACACACTCCTTTG |
| EGF | sense | TGTCCACGCAATGTGTCTGAA |
|  | antisense | CATTATCGGGTGAGGAACAACC |
| MMP2 | sense | TACAGGATCATTGGCTACACACC |
|  | antisense | GGTCACATCGCTCCAGACT |
| MMP9 | sense | AGACCTGGGCAGATTCCAAAC |
|  | antisense | CGGCAAGTCTTCCGAGTAGT |
| ENG | sense | GCATCCTTCGTGGAGCTACC |
|  | antisense | GAGGAGTGGTCTGGATCGG |
| PDGFA | sense | CCCCTGCCCATTCGGAGGAAGAGA |
|  | antisense | TTGGCCACCTTGACGCTGCGGTG |
| PDGFB | sense | CTCGATCCGCTCCTTTGATGA |
|  | antisense | CGTTGGTGCGGTCTATGAG |
| PDGFC | sense | GACTCAGGCGGAATCCAACC |
|  | antisense | CTTGGGCTGTGAATACTTCCATT |
| ISH probe sequences |  |  |
| lncRNA UBE2CP3 |  | TGTGTCACTAGGCATTGT |
