# Supplementary figures and images for: Long non-coding RNA UBE2CP3 enhances HCC cell secretion of VEGFA and promotes angiogenesis by activating ERK1/2/HIF-1α/VEGFA signalling in hepatocellular carcinoma
Source: J Exp Clin Cancer Res. 2018 Jun 4;37:113. doi: 10.1186/s13046-018-0727-1 (PMC5987644; doi:10.1186/s13046-018-0727-1)

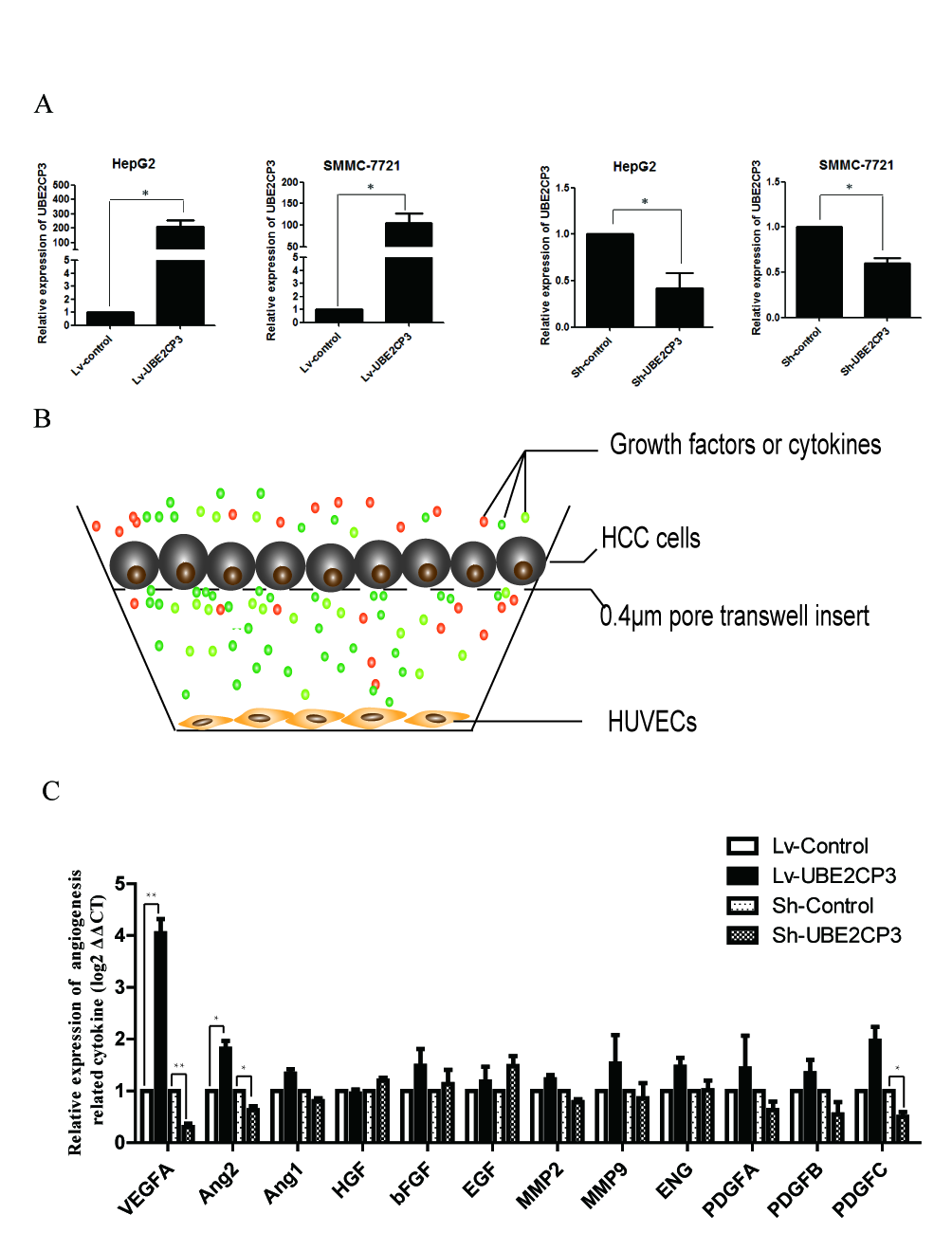

Supplement: Supplementary file 2 — Figure S1. (A) The infection efficiencies of lncRNA UBE2CP3 in HepG2 and SMMC-7721 were screened by qRT-PCR. (B) The schematic diagram for the co-culture system. (C) 12 kinds of common angiogenic factors were detected by qRT-PCR.. (TIF 24 kb) [file 13046_2018_727_MOESM2_ESM.tif]
